# Address

**Published:** 1868-08

**Authors:** W. F. Morrill


DENTAL REGISTER.

Vol. XXII.] AUGUST, 1868. [No. 8.

### Original Communications.

### ADDRESS

Read before the Indiana State Dental Association,

BY DR. W. F. MORRILL.

*Gentlemen:* By constitutional limitation the term of office to which you elected me now expires. In retiring from this honorable position, it is no affectation to say, that for your manifestations of kindness and indulgence toward me during our deliberations here, I tender you, most heartily, my acknowledgements. For these courtesies and the distinguished honor paid me, I must ever cherish as among the bright recollections of my life.

The regularly recurring of these assemblages, so replete with social and professional reunion, must ever warm our hearts with generous emotions, and awaken within us a renewed spirit, energy and force, to the duties of our honored vocation. Each one of them will indicate, like mile-stones, the progress of each mind, or, like tomb-stones, will mark the waste and decay, leaving to each of us a knowledge and consciousness of his condition. By mingling together here, by a free, hearty interchange of opinions, and by the love we bear toward our chosen profession, we are furnished with ample incentives to act, all that is possible for us. Most happily then these annual returns meet the present exigencies. Those who are here to-day come cheerfully from their fields of investigation, and generously lay their offerings before us for our examination and impartial scrutiny. Whatever we have let us then communicate with alacrity that the interests and business of this Association may be materially enhanced, and the means we have sought to discipline us for the more active duties of our profession shall be adequately realized. If this be done, our each annual return will be as a New Year's day. It will be an epoch that marks with faithful accuracy the development, progress, and achievements of our profession. It will be a calendar complete — a cycle of advancement finished — another opening to us in perspective.

This much preliminary to my subject;

Professional Character.—As a condition indispensable to the practice of Dentistry, in its refined and enabling perfections, professional character stands first and foremost. Just in proportion as its weight, dignity, and force are impressed on the world, just in the same degree will be felt the respect and admiration due to it. To possess the estimable qualities that go to make professional life wield a potency and power, unshadowed by blemishes, demands a vigilence and circumspection such as a few mortals only are permitted to share. Nevertheless, while we make Dentistry our calling, it is our prerogative to aim well at the mark, in order that we may not fall too far below a just standard of professional eminence.

We take our place in the world classified as among the liberal professions; we ask public recognition of our worth. It is expected of us that we will demonstrate that we *are* something as well as *know* something. Were we to look over the field of Dental practitioners, and carefully note those who reach above mediocrity — who are the bright particular stars of professional character — we should find the number comparatively small. Some of the hindrances to these nobler attainments in professional character, together with some suggestive hints to a fuller development of it, will be all I shall attempt at this time.

Frowning over the gate of our professional life, are the talismanic words, — "Power is the source of knowledge, and character the condition of intelligence." Professional success does not primarily depend on qualities strictly scientific and intellectual, but personal and constitutional. It's chief test is influence. It is the power of guiding, controlling, shaping other minds. It is exhibited in the ordinary affairs of the world as well as in the world of ideas. It is a force that is felt precisely as effect follows a cause. It can not be more fitly expressed than to say that it is not so much the profession that makes the Dentist as the Dentist the profession! Fine examples, living and dead, illustrate this fact. Take the lamented Harris or Townsend as an illustration; they were the impersonations of professional character, their opinions are authorities. The principles of our profession were inwrought into their natures, and carried with them the very instincts of life.

It is a fact, patent to most of us, that the practice of Dentistry is no bed of roses on which we may recline in luxurious ease; but antithesis to this, it is often beset with trials and difficulties quite hard to be borne, except by hearts heroic enough to manfully combat and surmount them all. In a word, we may say, to practice our profession, successfully, requires pluck. It is but natural for us to build up hopes, to cherish fond expectations for our calling, and by contact and friction with the world, see them perish. Day by day we are closely housed; we but seldom partake of sunlight or the pure air of heaven; we apply ourselves with unvarying diligence to the immediate affairs of our offices, and is it but natural that some of us should partake occasionally of a leaden sky, and now and then only catch a few faint gleams of the silver cloud-linings?

Such a life does not all the while best develope man, either physically or socially, to his nobler perfections. We are schooled in the agonies, pains, and complaints of other people. Do these lessons teach us to be jolly, mirthful, and sweet-tempered? Or are there occasional instances of gloomy, morose, sullen, forbidden natures, who have exhausted their sympathies and blended their dispositions with the character of their operations?

The public is a quick censor to detect character. How would any of us like to see the gauge wherewith we are graduated by public sentiment? How often do we reflect that, while in the discharge of the duties of our offices, we reveal, we make and unmake character? Patients, receiving our advice or services, place us in the balances of public sentiment, we are weighed and properly registered. Are you curious to know what the entry is? Be guarded, be mindful of what you say, be chary of promises, be courteous, be truthful, underlying and interwoven in all the conversations we have with patients, and in the advice we proffer them, must be honesty and truth. No one can doubt this, or hope to succeed in building up any strength of professional character without these cardinal virtues, and if thus favored, the grievous sins of ignorance and incompetence are not half so likely to return and plague us, as if the contrary were our condition.

Chief among the parasites that afflict the body professional is conceit. It is a pompous little animal for so much mischief. It sometimes burrows deep. It is full of bray and strut. It feeds on ignorance and soft blandishments of speech. It is seldom without wings. It loves to soar away into those dizzy realms of accomplishments, of scientific knowledge, of dextrous manipulative art, that the modest, meritorious, and meek gaze at its wondrous flights with marvelous amazement and due reverence. It is thus that some of the body professional are sometimes grieved. They always count upon successes, and never on failures. The former they sound as if with a Gabriel's triumph, the latter knows no mention. They cherish idols of their own. They seek no criticism, but proffer much. It is not unlikely if some of this class of Dentists were supplied with what Sidney Smith termed a "foolometer to warn them of dangers," it would not be an unwise nor insignificant suggestion.

Another hindrance in the pathway to professional character is a hobby. It is twin brother to conceit. "Hobbies," says Bulwer, "should be wives, not mistresses. It will not do to have more than one at a time. One hobby leads you out of extravagance; a team of hobbies you can not drive until you are rich enough to find corn for them all. Few men are rich enough for this."

Nor is it unfrequent that instances of genius crop out among us in professional character. Were we in doubt as to this fact we have but to step into the office of Dr. So and So, "Practical and Scientific Dentist," witness the wonderful mechanisms hanging around and embellishing his apartments. We patiently listen to the embryo discoveries of which he but faintly and slyly hints he is incubating, and in the process of time will astound the incredulous world. "Lo! he put in his thumb, pulled out a plum, and cried, what a great boy am I."

The charm of the patent office seal goes on to their every bantling not strangled at the parturition period, for avarice is their highest conception of the liberal profession wherewith they are supposed to be called. This arrogating to one's self the gifts of genius, or all the best methods of manipulative art, or knowledge of all the sciences pertaining to Dentistry, or to be a complete humbug generally, is supreme nonsense, and does positive harm in any efforts to make character. Besides, it is unworthy and illiberal toward associates in the profession; begets a weakened public confidence that will take a long time to correct and overcome.

If we would properly comprehend the profession to which we belong our hearts must morally and mentally reach out beyond personal interests. Nor does it lie in the conception of bigots to see "the grasp of beauty in benificence, the grandeur of truth told, and the majesty of right proclaimed."

Our Dental literatures is an index and reflex of professional character. Some of the contributions are quite valuable, and others exhibit such an elementary strain as to be quite successful in that direction; or such a verbosity of psychological and metaphysical essences that ordinary finite minds have some difficulty in comprehending the transcendent altitudes in which the writers are pleased to roam. The tone, the temper, and the completeness of that knowledge wherewith the contributors should be possessed, have not at all times characterized their productions. A proper spirit, a just measure of courtesy to those having new theories and new ideas, is the right way to receive them. To fly off in a tangent, to be denunciatory, to affect superior wisdom, are alike fatal to argument, and good sense. We are all seekers after truth; we desire nothing more—nothing less.

Look at the controversy going on between our microscopic friends with regard to the "inter globular theory." Are they contending for truth or victory? Both parties in the positions taken are equally sanguine and positive. Do they manifest the humble spirit that an English philosopher once did? Says he, "for my part, as well convinced as I am that two and two make four, if I were to meet with a person of credit, candor and understanding, who should seriously question it, I would give him a patient hearing." As pertinent to our microscopic friends, and a fine instance of character worthy of imitation I commend it. Permit me in further elucidation of this point to quote a case from Bulwer, who must have beheld our microscopic brethren when he says: "Some philosopher, let us suppose, had arrived at the conclusion that the moon is incapable of harboring organic life, ten persons whose evidence he just accepted in a matter on no pride of science is involved, tell him they have been looking through the telescope and they, one after another, have seen an enormous creature endowed with organic life—they entreat the philosopher to come and see this phenomenon himself—would the philosopher be justified in saying, 'I have satisfied myself that no such creature can possibly exist in the moon. It is within the laws of nature that you ten gentlemen should tell a falsehood, or be deceived by an optical illusion.' Would a philosopher be justified by any affection for truth in saying this? Certainly not." Sir Isaac Newton was wont to observe "It may be so; there is no arguing against facts and experiments." "He might indeed have beheld the monster whose existence destroyed his theory; but discovered on careful scrutiny that it was no inhabitant of the moon, but a blue-bottle fly that got on the lense and viewed through the telescope seemed bigger than a dragon." Our microscopic friends may take warning at this analagous case, lest their "balsamic mountings" should prove to be bigger than a dragon. And we may likewise remember that no theory, however well supported by authorities, and bearing the must of age on them, but are liable to be punctured and exploded by new revelations under the guidance of superior intelligence and patient research. Moreover it is impossible to creep stealthily into the minds of others wielding a large influence, a large brain, and use what you can to give currency to your own sentiments, thereby assisting you up into an atmosphere congenial to you but noxious to others.

Perhaps, the most melancholy spectacle of a failure in professional life and character, is a colaborer grown gray in service, but who manifests no disposition to rise above the petty jealousies that environ him and acknowledge with frankness and candor the claims of his associates of conceded abilities. He no doubt occasionally feels the ominous glance of his own accusing eye; and, accordingly, he intrenches himself behind bits of scandal, detraction, suspicions, sneers and defences of that sort. His pusillanimous soul has never been emancipated from malice and all uncharitableness. From the great lights of the profession he shrinks away into his kennel, and is cowed by "swift sweep of a daring will." Nothing but a series of reconstruction acts will save him, and perhaps not even these.

Were I to counsel the young members of our profession, I would in the words of Thackery say: "Shun the society of such men; frequent the society of your betters. Learn to admire rightly. Note what the best skilled admire: they admire things of value. Narrow spirits admire basely and worship meanly." Pursuing a course like this you are in the true process for elevating and refining professional character.

Behavior and conduct while in the presence of patients reveal the claims we may have to the true dignity of manhood, and a just appreciation of professional merits. Harshness of temper, rude and imperious manners, garrulous nothings, stupid sympathies, in any or all of these, while endeavoring to control a patient, will neither further your confidence nor respect. Have a presence that commands obedience. A confidence inspired in this way will half disarm dental operations of their terrors. Of course, no dignity however well put on and concealed will avail you anything, unless backed by an intelligent and thorough knowledge of your profession, and then not always. A comprehension of our professional character presupposes good habits; and by these I mean those above reproach. Too much time should not be frittered away in sports, nor amusements, nor street-corner confabs. They add nothing to any one's stock of information. They will not keep your office, nor will your office keep you. Cultivate the sweet amenities of life. Preserve an even temper, especially even, when a tempest in a tea-pot comes boiling at your door in the shape of an old woman who tells you in unmistakable terms that *her teeth don't fit.* Nor should you so far forget the honor and true welfare of your profession, if, occasionally, you be reminded by extravagant advertisements of the Drs. Pluguglies and

Drs. Tushmakers, that all the teeth in your neighborhood are being "pulled by gas," and other startling exploits performed for very moderate sums in greenbacks; you cannot improve your acquaintance by any notice of these "bummers" whatever. Let them severely alone. You have character to preserve, they have none to lose.

Finally, we have reason to congratulate ourselves that the standard of professional character is steadily rising higher in our own and in public estimation. Since the adoption of our code of ethics, we still hope for it brighter and better things.

As an additional prop to this we now need legislative enactments such as shall protect the weak and ignorant from the hands of mountebanks now quartered in every community, and which shall reward merit over incompetency. Could the people of Indiana seriously, as we do, realize the full importance of this measure, there would be no hesitancy, but great alacrity in hastening the passage of such a benificent law.

Let us then come square up to the full dignity of our manhood, lend whatever influence we have, give our time and money in aid, and furtherance of every object best calculated to further the real and true interests of the profession to which we have consecràted ourselves. Do this, and the golden gleams from yonder mountain of professional character you so much covet shall shine upon you with a solar radiance.

### "PEACE, RUDE BOREAS."

Editors Dental Register.—*Gentlemen:* — It will, ere this reaches you, have been marked, by those of your readers having the requisite intelligence, that the comments in your journal, upon my remarks on our knowledge of Dental tissues, entirely misapprehend, or else misrepresent,—intentionally misrepresent,—their character. I can not, I confess, see otherwise than the latter, for my meaning was plain, —very plain.

